# Circular RNA profiles and the potential involvement of down‐expression of hsa_circ_0001360 in cutaneous squamous cell carcinogenesis

**DOI:** 10.1002/2211-5463.13114

**Published:** 2021-03-11

**Authors:** Pingjiao Chen, Changxing Li, Hongchang Huang, Liuping Liang, Jing Zhang, Qian Li, Qi Wang, Sanquan Zhang, Kang Zeng, Xibao Zhang, Jingyao Liang

**Affiliations:** ^1^ Department of Dermatology Nanfang Hospital Southern Medical University Guangzhou China; ^2^ Forevergen Biosciences Guangzhou China; ^3^ Institute of Dermatology Guangzhou Medical University China; ^4^ Department of Dermatology Guangzhou Institute of Dermatology China

**Keywords:** circular RNA, cutaneous squamous cell carcinoma, expression profile, hsa_circ_0001360

## Abstract

Circular RNAs (circRNAs) act as sponges of noncoding RNAs and have been implicated in many pathophysiological processes, including tumor development and progression. However, their roles in cutaneous squamous cell carcinoma (cSCC) are not yet well understood. This study aimed to identify differentially expressed circRNAs and their potential functions in cutaneous squamous cell carcinogenesis. The expression profiles of circRNAs in three paired cSCC and adjacent nontumorous tissues were detected with RNA sequencing and bioinformatics analysis. The candidate circRNAs were validated by PCR, Sanger sequencing and quantitative RT‐PCR in another five matched samples. The biological functions of circRNAs in SCL‐1 cells were assessed using circRNA silencing and overexpression, 3‐(4,5‐dimethylthiazol‐2‐yl)‐5‐(3‐carboxymethoxyphenyl)‐2‐(4‐sulfophenyl)‐2H‐tetrazolium inner salt (MTS), flow cytometry, transwell and colony formation assays. In addition, the circRNA–miRNA–mRNA interaction networks were predicted by bioinformatics. In summary, 1115 circRNAs, including 457 up‐regulated and 658 down‐regulated circRNAs (fold change ≥ 2 and *P* < 0.05), were differentially expressed in cSCC compared with adjacent nontumorous tissues. Of four selected circRNAs, two circRNAs (hsa_circ_0000932 and hsa_circ_0001360) were confirmed to be significantly decreased in cSCC using PCR, Sanger sequencing and quantitative RT‐PCR. Furthermore, hsa_circ_0001360 silencing was found to result in a significant increase of the proliferation, migration and invasion but a significant decrease of apoptosis in SCL‐1 cells *in vitro*, whereas hsa_circ_0001360 overexpression showed the opposite regulatory effects. hsa_circ_0001360 was predicted to interact with five miRNAs and their corresponding genes. In conclusion, circRNA dysregulation may play a critical role in carcinogenesis of cSCC, and hsa_circ_0001360 may have potential as a biomarker for cSCC.

## Graphical abstract

The expression profiles of circular RNAs (circRNAs) were analyzed using high‐throughput RNA sequencing between paired cutaneous squamous cell carcinoma and adjacent nontumorous tissues. In total, 1115 circRNAs (457 up‐ and 658 down‐regulated circRNAs) were differentially expressed in cutaneous squamous cell carcinoma. Down‐regulation of hsa_circ_0001360 significantly increased proliferation, migration and invasion but decreased apoptosis of SCL‐1 cells *in vitro*, whereas up‐regulation of hsa_circ_0001360 had the opposite effects.

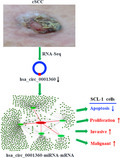

**Abbreviations**
circRNA: circular RNA
cSCC: cutaneous squamous cell carcinoma
DMEM: Dulbecco's modified Eagle's medium
gDNA: genomic DNA
GO: Gene Ontology
KEGG: Kyoto Encyclopedia of Genes and Genomes
MRE: miRNA response element
PHC3: polyhomeotic homolog 3
qRT‐PCR: quantitative RT‐PCR
RNA‐seq: RNA sequencing
SCL‐1 cell: human squamous carcinoma cell line
siRNA: small interfering RNA

Cutaneous squamous cell carcinoma (cSCC) is the second most common nonmelanoma skin cancer after basal cell carcinoma, accounting for >20% of skin cancers, with an increasing annual incidence worldwide [1, 2]. cSCC generally develops more rapidly and is more inclined to metastasize into regional lymph nodes and distant sites or recur locally than basal cell carcinoma [3, 4]. Although most cSCC is cured by adequate surgery and/or radiotherapy, more than 2.0% still die of the disease, similar to deaths from renal and oropharyngeal carcinomas, and melanoma in the Southern and Central United States [5]. Recurrence rates of cSCC are usually 3–8% for all cSCCs [6], whereas 5‐year recurrence rates of advanced cSCC are high at between 36% and 63% [1, 7]. Once recurrence of cSCC has occurred, patients have a much worse prognosis [1]. The reported 10‐year survival rate is <20% in patients with regional lymph node metastasis and <10% in patients with distant metastasis [8, 9]. Thus, there is an urgent need to develop novel effective targets/biomarkers and therapeutic approaches for the treatment of cSCC.

The occurrence and development of cSCC is a complex pathological process involving a series of factors and mechanisms. Cumulative sun/other ultraviolet radiation exposures were found to be the most important environmental risk factors by causing DNA damage of keratinocytes in white individuals [10]. However, scarring processes and inflammatory conditions (e.g. burn scars and chronic ulcers) were considered as important etiologic factors in black and Asian individuals [10, 11]. Patients with recessive dystrophic epidermolysis bullosa showed a significant 50‐fold increased incidence of cSCC, which is independent of ultraviolet exposure [12]. Transplant immunosuppression increases the risk for cSCC by 65–250 times and tends to be more aggressive in organ transplant recipients compared with the general population [13]. A number of key gene alterations have been shown to be involved in the pathogenesis and development of cSCC, such as tumor suppressors (*p53*, *CDKN2A*, *p16*, *KMT2D*), cell proliferation/apoptosis/cell signaling (*NOTCH*, *PI3K/AKT*, *RAS/MAPK*, *INPP5A*, *EGFR*, *CYFIP1*, *E6*, *TERTp*), immune system regulations (*PD‐L1*, *lectin‐like transcript 1*), tissue invasion (*E‐cadherin*, *podoplanin*, *chemerin*, *FAK*) and epigenetic regulation (*KMT2C*, *ERRα*, CpG promoter methylation) [1, 14, 15]. In addition, accumulating studies have found that miRNAs play a significant role in various human cancers, including cSCC, which act as oncogenes or tumor suppressors through down‐regulation of multiple target genes at the post‐transcriptional level [16, 17]. For example, miRNA‐124 [18] and miRNA‐34a [17] were significantly down‐regulated in cSCC tissues/cell lines and were inversely correlated with the aggressive progression of cSCC. miRNA‐365 and miRNA‐21 were two of the highest expressed miRNAs in response to UVB irradiation treatment [19] and were markedly expressed in clinical specimens of cSCC [20, 21]. miRNA‐365 was identified as an upstream effector of p53 [4], and miRNA‐34a was a downstream effector of p53 [22], which can serve as a potential therapeutic biomarker of cSCC. miRNA‐21 via the phosphatidylinositol 3‐kinase (PI3K)/AKT/mammalian target of rapamycin (mTOR) signaling pathway [20] and miRNA‐193b/365a through the RAS/mitogen‐activated protein kinases (MAPK) signaling pathway [23] participated in the cSCC proliferation, apoptosis, migration, invasion and tumorigenesis.

Recently, circular noncoding RNAs (circRNAs), covalently closed circular structures of noncoding RNAs, were reestablished as a new star of RNA word in eukaryotes [24]. By acting as a miRNA sponge, circRNAs can regulate alternative splicing and transcription and modulate the target gene expression [25, 26]. Moreover, circRNAs also can serve as sponges for the RNA binding proteins and regulate their activity or recruit the components of RNA–protein complexes to affect the expression of target genes [25, 27]. circRNAs are widely expressed in a variety of tissues and evolutionarily conserved across the eukaryotic tree of life [28]. circRNAs are present predominantly in cytoplasm and are highly stable due to their remarkable resistance to RNA exonuclease and RNase [25]. So far, more than 3000 circRNAs have been identified from various species using high‐throughput RNA sequencing (RNA‐seq) [29]. Similar to miRNAs, circRNAs are expressed aberrantly and are involved in the pathogenesis of many diseases, including tumor, and may be promised as new diagnostic/therapeutic biomarkers [30]. However, the biological function and molecular mechanism of circRNAs underlying cSCC are still largely unknown [26, 31, 32]. This study investigated the expression profiles of circRNAs in cSCC tissue by high‐throughput RNA‐seq and bioinformatics analysis, and identified the tumor's suppressor role of hsa_circ_0001360 in progression of SCL‐1 cells.

## Results

### Differential circRNA expression profiles in cSCC

**Fig. 1:**
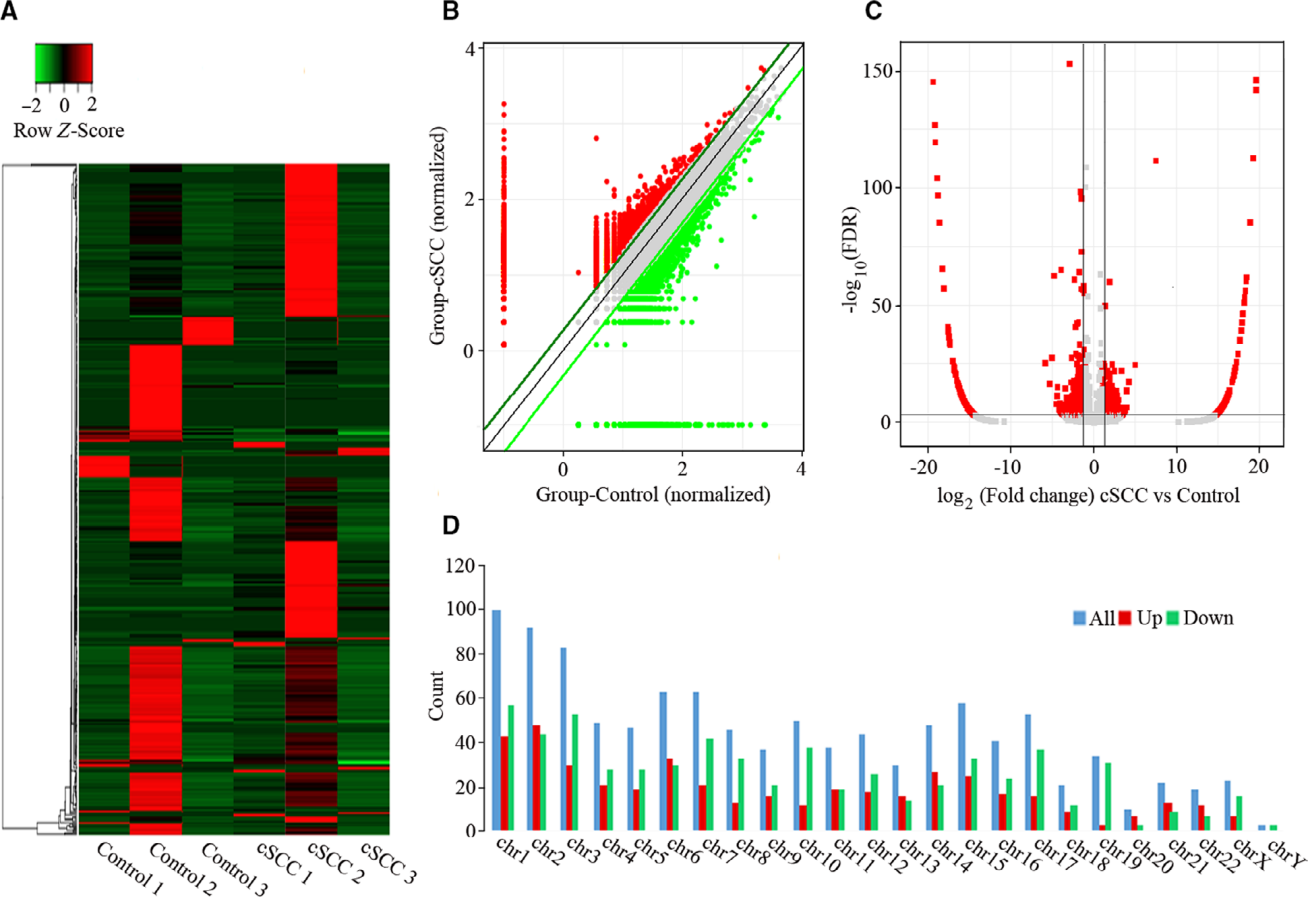
Differences and characterizations in circRNA expression profiles between cSCC and adjacent nontumorous tissues. (A) Hierarchically clustered heatmap analyzed circRNA expression profiles of the six samples, with columns representing samples and rows representing circRNAs. Up‐regulation was shown in red, and down‐regulation was in green. (B) Scatterplots displayed the differential circRNA expression between cSCC (*y* axis) and adjacent nontumorous tissues (*x* axis). The circRNAs above the top green line and below the bottom green line indicated more than 2‐fold change of circRNAs between the two groups of compared samples. (C) Volcano plots represented the differential circRNA expression between cSCC and adjacent nontumorous tissues. The red points in the plot represented the differentially expressed circRNAs with statistical significance. (D) Chromosomal distributions of differentially expressed circRNAs.

To investigate the abnormal expression of circRNAs, we performed high‐throughput RNA‐seq technology and bioinformatics analysis in three pairs of cSCC and adjacent nontumorous tissues. The results of hierarchical clustering exhibited the distinguishable circRNA expression profiles among six samples (Fig. 1A). The scatterplot and volcano plot displayed the significant differences in circRNA expression between cSCC and adjacent nontumorous tissues (Fig. 1B,C). Moreover, the differential expression circRNAs were shown to be widely distributed on all chromosomes, among which the top five chromosomes were chr1, chr2, chr3, chr6 and chr7 (Fig. 1D). Finally, a total of 1115 circRNAs were found to be differentially expressed in cSCCs compared with adjacent nontumorous tissues (fold change > 2.0, *P* < 0.05), of which 457 circRNAs were up‐regulated and 658 circRNAs were down‐regulated in tumor tissues.

### Validation of the differentially expressed circRNAs

**Fig. 2:**
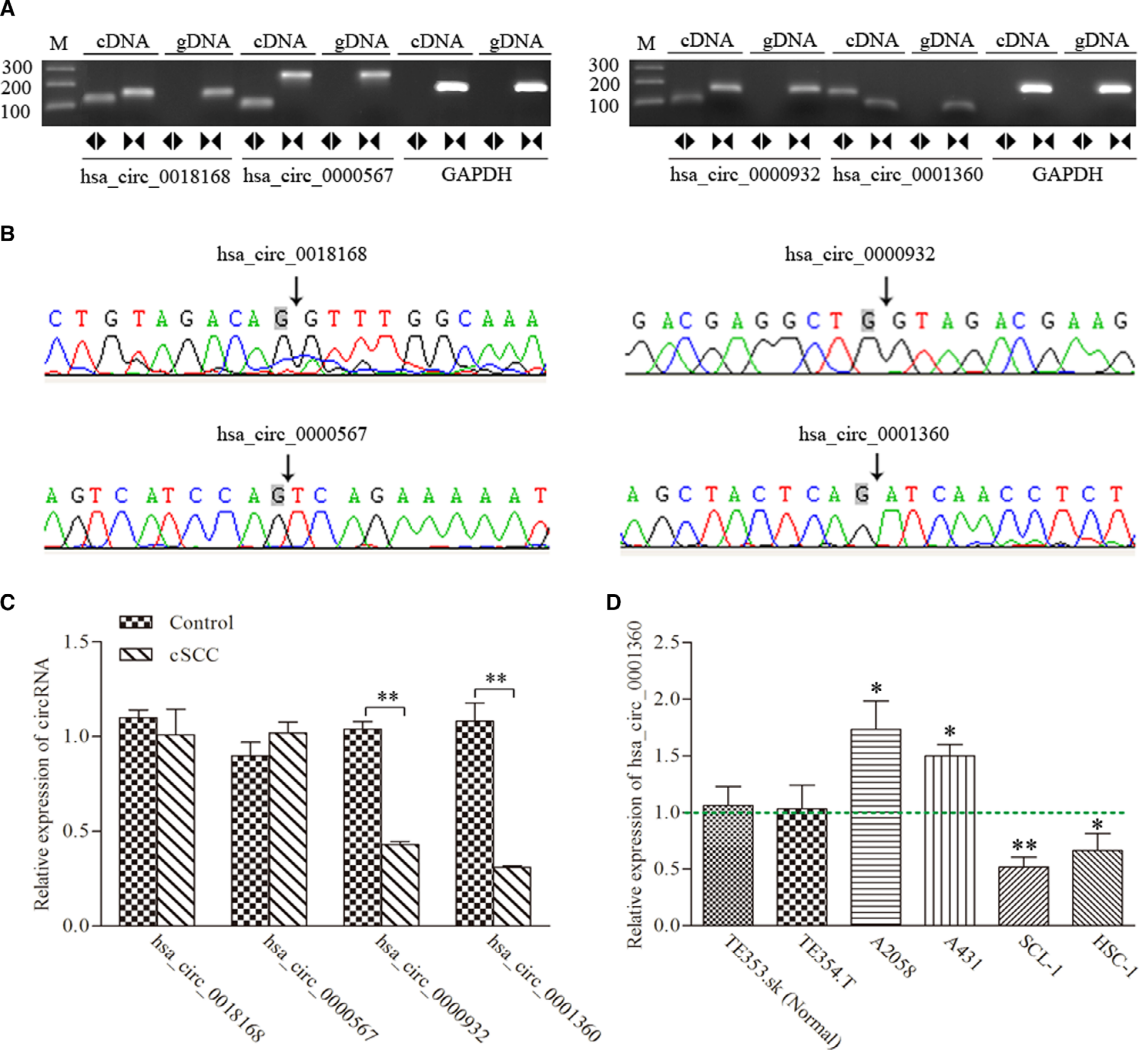
Identification of four candidate circRNAs. (A) RT‐PCR with divergent (circular) and convergent (line) primers was used to confirm the candidate circRNAs in samples of cSCC tissues. Divergent (circular) primers (◄►) can amplify a single fragment at the expected sizes in cDNA, but not in gDNA. Convergent (line) primers (►◄) can amplify a single fragment at the expected sizes in cDNA and gDNA. (B) Sanger sequencing of the candidate circRNAs showed the backsplice junction. (C) qRT‐PCR showed the expression levels of the four candidate circRNAs between cSCC and adjacent nontumorous tissues. (D) qRT‐PCR showed the expression levels of hsa_circ_0001360 in TE353.sk, TE354.T, A2058, A431, SCL‐1 and HSC‐1 cell lines. Student's *t*‐test and one‐way ANOVA were used. The data were expressed as mean ± standard deviation (*n* = 3). *P < 0.05, **P < 0.01 represented statistical difference.

To validate the RNA‐seq results and investigate the potential functions, we selected the typical differential expression circRNAs, including one up‐regulated circRNA (hsa_circ_0000567) and three down‐regulated circRNAs (hsa_circ_0018168, hsa_circ_0000932, and hsa_circ_0001360), for verification experiments in another five paired samples of cSCC and adjacent nontumorous tissues. RT‐PCR showed that a single fragment at the expected size of each of the four selected circRNAs was successfully amplified from cDNA, but not genomic DNA (gDNA), using divergent (circular) primers (Fig. 2A). Furthermore, the sequence of head‐to‐tail splice junctions of each circRNA was confirmed directly by Sanger sequencing of PCR amplicons (Fig. 2B). Consistent with the findings of RNA‐seq data, the results of quantitative RT‐PCR (qRT‐PCR) showed that two selected circRNAs (hsa_circ_0000932 and hsa_circ_0001360) are significantly down‐regulated in tumor tissues (Fig. 2C). However, the remaining two selected circRNAs were not statistically differentially expressed. In addition, the expression levels of hsa_circ_0001360 were further investigated in the different cell lines, including SCC cell lines (HSC‐1 and SCL‐1), basal cell carcinoma (BCC) cell lines (A2058 and A431) and a human normal skin cell line (TE353.sk). Quantitative PCR results demonstrated that the expression levels of hsa_circ_0001360 were lower in SCL‐1 and HSC‐1 cell lines, but higher in A2058 and A431 cell lines, as compared with TE353.sk. The SCL‐1 cell line was chosen for further study because it showed the lowest expression levels of hsa_circ_0001360, similar to the results of tissues obtained from five patients with cSCC (Fig. 2D).

### hsa_circ_0001360 regulated proliferation and apoptosis of SCL‐1 cells

**Fig. 3:**
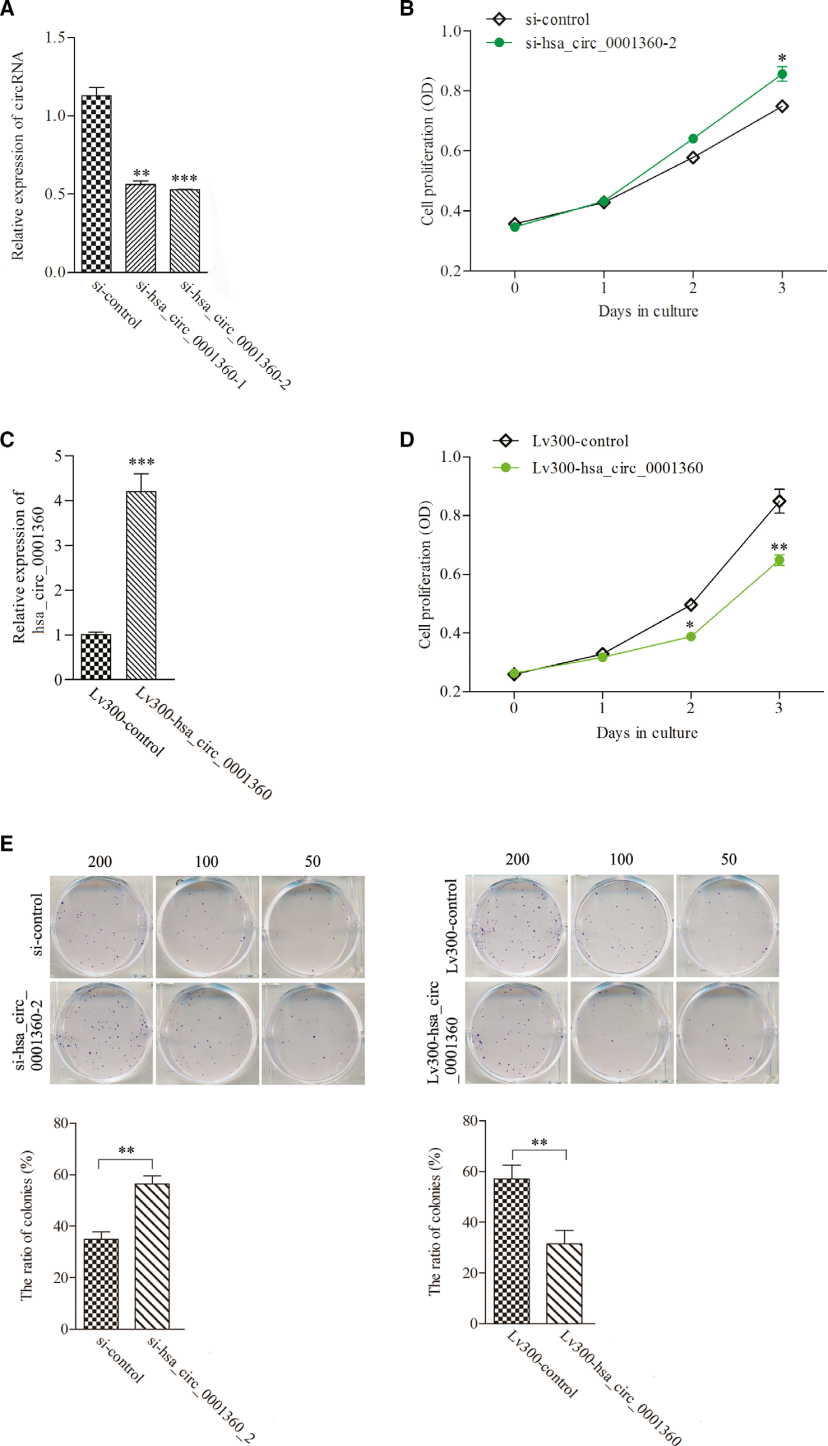
hsa_circ_0001360 regulated proliferation and colony formation activity of SCL‐1 cells. (A) The expression levels of hsa_circ_0001360 were significantly decreased after transfection with specifically synthesized siRNA in SCL‐1 cells. (B) The proliferation activity of SCL‐1 cells was significantly increased after transfection with si‐hsa_circ_0001360‐2. (C) The expression levels of hsa_circ_0001360 were significantly up‐regulated after transfection with Lv300‐hsa_circ_0001360‐2. (D) The proliferation activity of SCL‐1 cells was decreased after transfection with Lv300‐hsa_circ_0001360‐2. (E) The colony formation activity of SCL‐1 cells was increased after transfection with si‐hsa_circ_0001360‐2 and was decreased after transfection with Lv300‐hsa_circ_0001360‐2. Student's *t*‐test and one‐way ANOVA were used. The data were expressed as mean ± standard deviation (*n* = 5). *P < 0.05, **P < 0.01, ***P < 0.001 represented statistical difference.

**Fig. 4:**
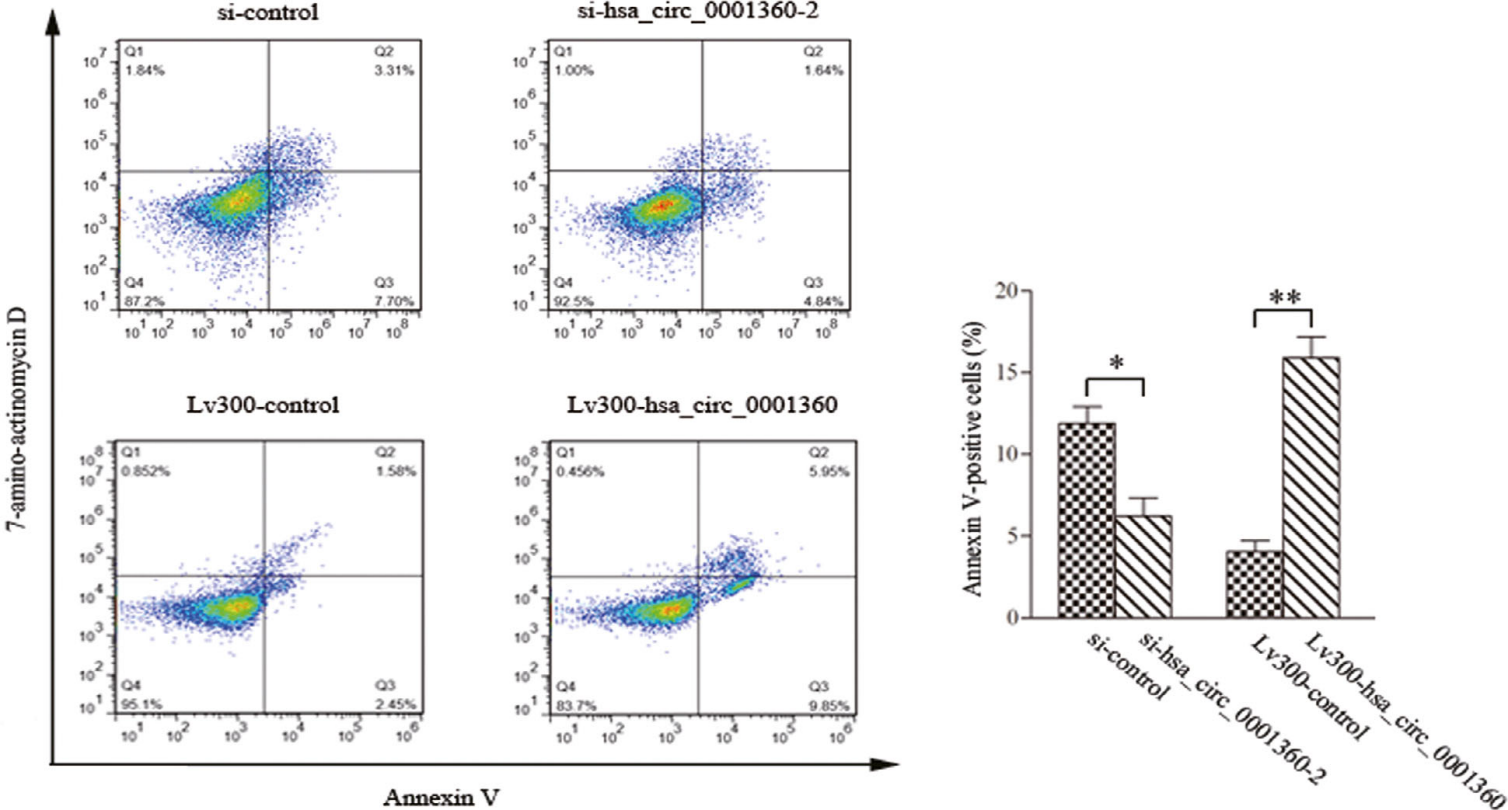
Flow cytometry assays showed that hsa_circ_0001360 regulated apoptosis in SCL‐1 cells. Student's *t*‐test was used. The data were expressed as mean ± standard deviation (*n* = 3). *P < 0.05, **P < 0.01 represented statistical difference.

The specifically synthesized small interfering RNAs (siRNAs) targeting hsa_circ_0001360 were transfected into SCL‐1 cells, which markedly down‐regulated the expressions of hsa_circ_0001360 compared with control cells (Fig. 3A). MTS assay revealed that cell proliferation was significantly enhanced by silencing of hsa_circ_0001360 (Fig. 3B). The full‐length cDNA of hsa_circ_0001360 was cloned into the specific vector, which was transfected and significantly increased the expression of hsa_circ_0001360 in SCL‐1cells (Fig. 3C). The proliferation curves showed that overexpressed hsa_circ_0001360 significantly attenuated SCL‐1 cell proliferation compared with the control vector (Fig. 3D). Similarly, silencing of hsa_circ_0001360 significantly increased the colony formation activity of SCL‐1 cells, whereas overexpressed hsa_circ_0001360 inhibited the colony formation activity of SCL‐1 cells (Fig. 3E). In addition, flow cytometry assays showed that silencing of hsa_circ_0001360 decreased apoptosis of SCL‐1 cells. On the contrary, overexpressed hsa_circ_0001360 could markedly increase the proportion of apoptotic cells (Fig. 4).

### hsa_circ_0001360 regulated the migration and invasion of SCL‐1 cells

**Fig. 5:**
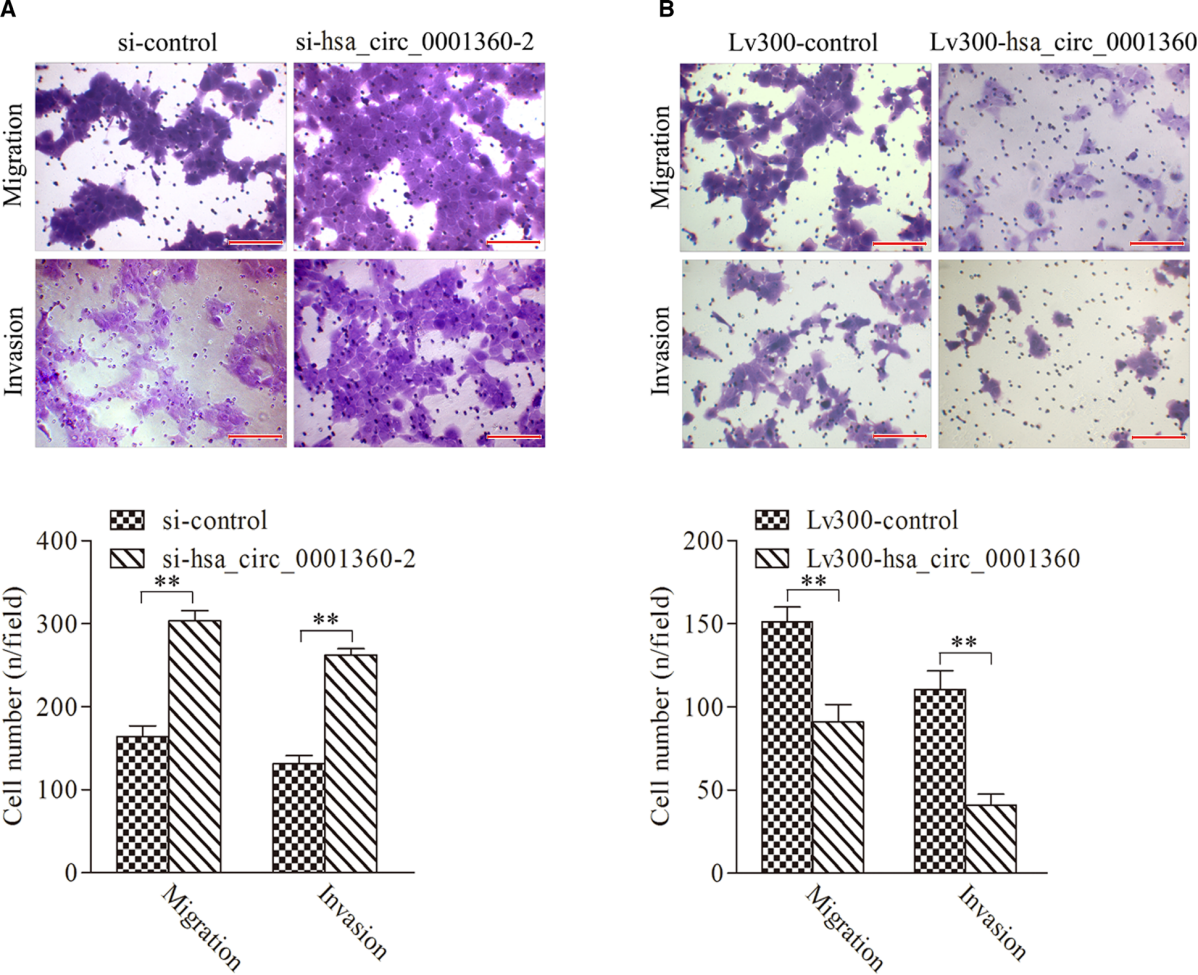
hsa_circ_0001360 regulated the migration and invasion of SCL‐1 cells. (A) Both the migration and invasion of SCL‐1 cells were significantly increased after transfection with si‐hsa_circ_0001360‐2. (B) Both the migration and invasion of SCL‐1 cells were significantly decreased after transfection with Lv300‐hsa_circ_0001360‐2. Scale bars represent 20 μm. Student's *t*‐test was used. The data were expressed as mean ± standard deviation (*n* = 5). **P < 0.01 represented statistical difference.

To explore the potential impact of hsa_circ_0001360 on migration and invasion of cSCC, we performed transwell assays in SCL‐1 cells. Results showed that silencing of hsa_circ_0001360 significantly enhanced the migration and invasion of SCL‐1 cells compared with control groups, whereas overexpression of circRNA‐0001360 caused the opposite effects (Fig. 5). These results indicated that circRNA‐0001360 might play an important role in the invasion and metastasis of cSCC.

### Prediction of circRNA–miRNA–mRNA interaction network of hsa_circ_0001360

**Fig. 6:**
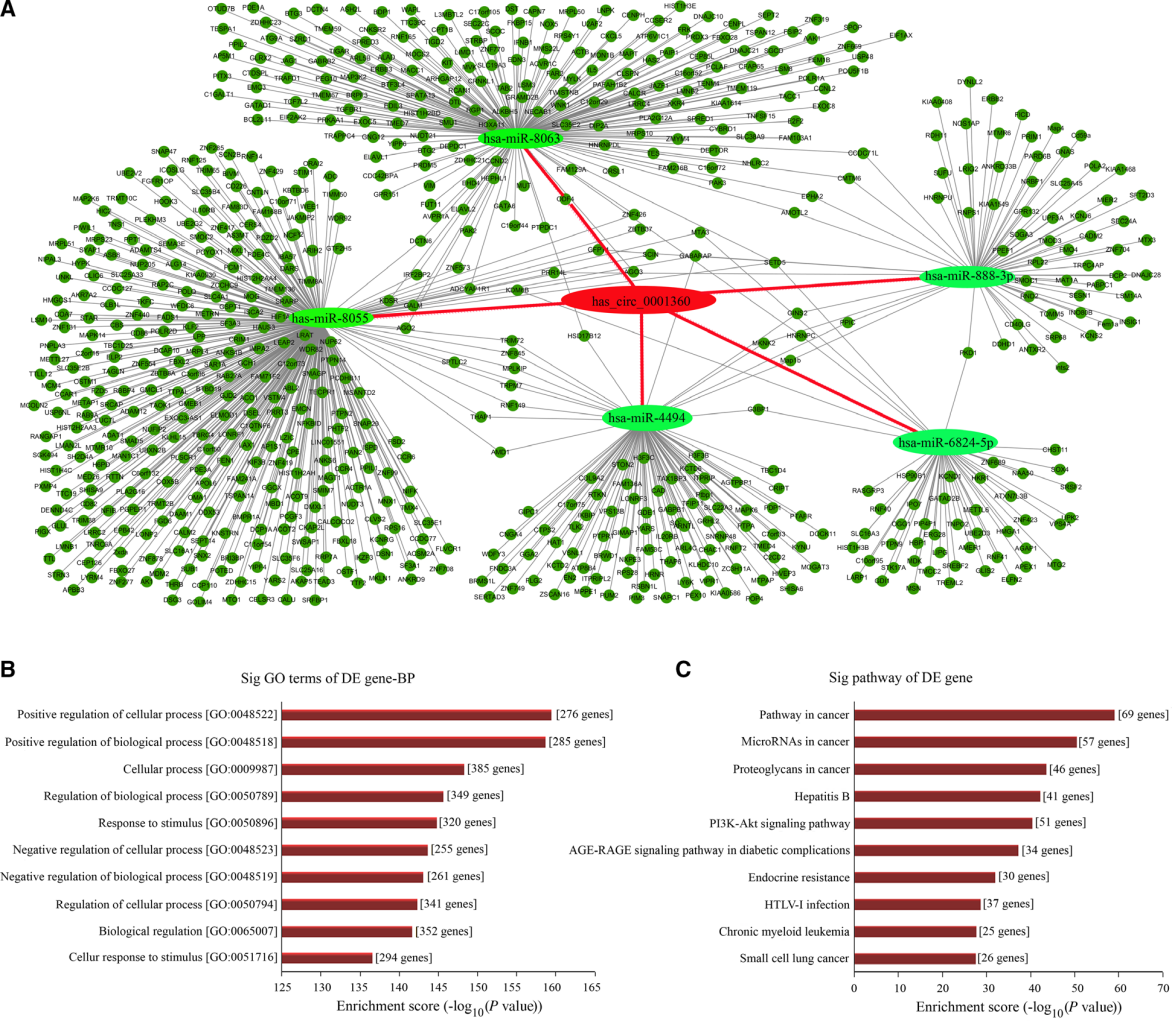
circRNA–miRNA–mRNA network, GO, and KEGG pathway analysis for hsa_circ_0001360. (A) The predicted hsa_circ_0001360 targeted the circRNA–miRNA–mRNA gene coexpression network. The five miRNAs (hsa‐miR‐8055, hsa‐miR‐8063, hsa‐miR‐4494, hsa‐miR‐888‐3p, and hsa‐miR‐6824‐5p) and their mRNA target genes were displayed based on sequence‐pairing prediction. (B) GO enrichment analysis for hsa_circ_0001360 in terms of biological processes (BP). The top 10 significantly enriched target genes and their scores (negative logarithm of *P* value) were listed as the *x* axis and the *y* axis, respectively. (C) KEGG pathway analysis for hsa_circ_0001360. The top 10 significantly enriched pathways and their scores (negative logarithm of *P* value) were listed as the *x* axis and the *y* axis, respectively.

Because circRNAs interacted with target miRNAs via miRNA response elements (MREs) and then regulated gene expression, the potential circRNA–miRNA–mRNA interaction network of hsa_circ_0001360 was predicted using TargetScan (http://www.targetscan.org/) and MiRanda (http://www.microrna.org/microrna/home.do) software. There are a total of 5 miRNAs, and 726 mRNAs were predicted to be targeted by hsa_circ_0001360 through its specific base, which was displayed using cytoscape analysis. As shown in Fig. 6A, hsa‐miR‐8055 exhibited the largest interaction network, followed by hsa‐miR‐8063, hsa‐miR‐4494, hsa‐miR‐888‐3p and hsa‐miR‐6824‐5p. Also, hsa_circRNA_0001360 was predicted to share complementary binding sites with hsa‐miR‐8055, hsa‐miR‐8063, hsa‐miR‐4494, hsa‐miR‐888‐3p and hsa‐miR‐6824‐5p based on MRE sequences (Table 1). To further analyze the functions of hsa_circ_0001360, we performed the Gene Ontology (GO) and Kyoto Encyclopedia of Genes and Genomes (KEGG) pathway analyses (Fig. 6B,C). GO analysis revealed that the significantly enriched target genes of hsa_circ_0001360 were strongly involved in terms of cellular process, biological process, response to stimulus and so on. KEGG analysis showed the top 10 significantly enriched pathways associated with the down‐regulated hsa_circ_0001360, including pathways in cancer, miRNAs in cancer, proteoglycans in cancer, hepatitis B, PI3K–Akt signaling pathway and so on.

**Table 1:**
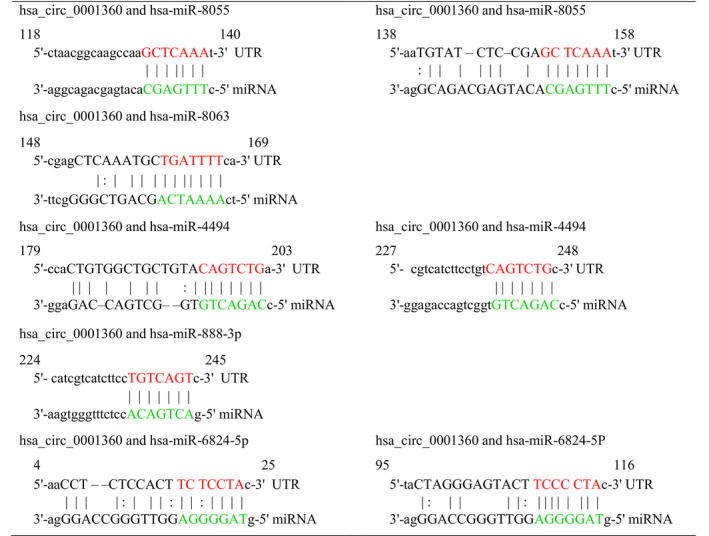
The MRE sequences of hsa_circ_0001360 and its target miRNAs.

## Discussion

Although dysregulation of specific circRNAs has been shown to play oncogenic and tumor‐suppressive roles in various cancers, only a few studies have reported the expression profiling and/or the carcinogenesis of circRNAs in cSCC [26, 31, 32]. In 2016, Sand *et al*. [26] first identified 322 differentially expressed circRNAs (143 up‐regulated and 179 down‐regulated) between three cSCCs and three nontumorous tissues using human circRNA microarray, but the functional roles of these candidate circRNAs have not been explored. Subsequently, based on these results of Sand *et al*. [26], the differentially expressed hsa_circ_0070934 was recently chosen for investigation of its biological functions and mechanisms in cSCC by another independent study [31], in which hsa_circ_0070934 was shown to be highly expressed and to promote cell proliferation and invasion by sponging miR‐1238 and miR‐1247‐5p in cSCC/cell lines. In addition, Verduci *et al*. [32] reported that circPVT1 was significantly up‐regulated through the mut‐p53/YAP/TEAD complex in patients with head and neck SCC and was involved in the control of cell proliferation through modulating the expression of miR‐497‐5p and genes. In this study, we found that a total of 1115 circRNAs, including 457 upregulated and 658 down‐regulated circRNAs, were aberrantly expressed in cSCC compared with adjacent nontumorous tissues using high‐throughput RNA‐seq. We further confirmed that hsa_circ_0001360 was significantly down‐regulated in cSCC tissue by qRT‐PCR, which was consistent with RNA‐seq data. Silencing hsa_circ_0001360 significantly increased the proliferation, invasion and migration, and decreased apoptosis of SCL‐1 cells. Conversely, overexpression of hsa_circ_0001360 caused the opposite effects. According to bioinformatics analysis, the hsa_circ_0001360/miRNAs (hsa‐miR‐8055, hsa‐miR‐8063, hsa‐miR‐4494, hsa‐miR‐888‐3p, and hsa‐miR‐6824‐5p)/mRNA (726 genes) may play a crucial role in cSCC progression through the PI3K/AKT and/or other oncogenic signaling pathways. However, only the SCL‐1 cell line was chosen for the functional experiments of hsa_circ_0001360, and further study using a larger panel of cSCC cell lines is required to confirm these findings.

Unfortunately, the biological roles of hsa_circ_0001360 are currently very little studied. Only a recent study [33] reported that hsa_circ_0001360 was markedly down‐regulated in the placental tissue and plasma of small‐for‐gestational‐age fetuses compared with appropriate‐for‐gestational‐age fetuses and had two target miRNAs (hsa_miR_4725_3p and hsa_miR_671_5p) that were significantly elevated in placental tissue of small‐for‐gestational‐age fetuses. Interestingly, hsa_miR_4725_3p was observed to be up‐regulated in patients with glaucoma [34] and was involved in xanthohumol‐attenuated glioma cell invasion [35] and cotreatment of melatonin‐ and pterostilbene‐induced apoptosis of colorectal cancer cells [36]. hsa_miR_671_5p is dysregulated in different human malignancies, acting as a tumor suppressor in the progression of esophageal SCC [37], osteosarcoma [38] and gastric cancer [39], or acting as an oncogene in oral SCC [40], colon cancer [41] and so on. hsa_circ_0001360 is transcribed from the parental gene polyhomeotic homolog 3 (PHC3), lying within a region of human chromosome 3q26.2. PHC3 is a ubiquitously expressed member of the polycomb complex that associates with cellular proliferation [42, 43]. PHC3 expression was absent in a majority of primary osteosarcoma tumors, and loss of PHC3 function would favor tumorigenesis by potentially disrupting the ability of cells to remain in G0 [42]. Coxsackievirus B3 infection can induce miRNA to inhibit the expression of PHC3 in HeLa cells and decrease binding of PHC3 to the transcription factor E2F6 as a polycomb complex that can silence promoters of target genes to prevent cell‐cycle progression[43]. In addition, PHC3 also was found to be frequently up‐regulated in three common epithelial neoplasms (lung SCC, uterine carcinoma and ovarian serous carcinoma), which may act as a potential oncogene [44]. Future molecular studies are required to carefully dissect the role of PHC3 in different human cancers.

In conclusion, this study first confirmed that hsa_circ_0001360 was down‐regulated in cSCC. The hsa_circ_0001360 overexpression inhibited cSCC cell proliferation, invasion and migration. hsa_circ_0001360 may play important roles in cSCC through sponging tumor‐associated miRNAs, which may be a new potential therapeutic target for cSCC.

## Materials and methods

### Study subjects and samples

This study was approved by the Medical Ethics Committee at Guangzhou Institute of Dermatology and Nanfang Hospital of Southern Medical University (Guangzhou, China), respectively, and all patients provided written informed consent for the use of surgical samples. The study conformed to the ethical standards of the World Medical Association Declaration of Helsinki. Tissue samples of approximately 1 g were collected immediately after cSCC resection. There was no targeted therapy, radiotherapy or chemotherapy before the surgery in the recruited patients. A total of eight cSCC tissues and paired adjacent nontumorous tissues (three for RNA‐seq analysis; five for PCR, Sanger sequencing and qRT‐PCR) were collected from patients in 2017. The cSCC tissues and adjacent nontumorous tissues that were taken about 5 cm from the edge of the cancer were confirmed by two independent pathologists. Fresh samples obtained through surgery were immediately submerged in RNAlater (Ambion, Austin, TX, USA) and frozen in liquid nitrogen until further use.

### Library preparation and Illumina sequencing

Total RNA was extracted from tissues using TRIzol (Invitrogen, Carlsbad, CA, USA). RNaseR treatment is performed to digest all the linear RNAs. The sample is purified with RNAClean beads, and the retrieved RNA is fragmented using divalent cations at an elevated temperature. Random hexamer primer is used to synthesize the first‐strand cDNA. Fragments are purified with AMPure beads and resolved in elution buffer (EB) buffer for end repair and adding A at the 3′ end. Y‐adaptor is added afterward. Then the product is amplified to construct the cDNA library. Finally, the quality of the libraries was assessed using Agilent 2100 Bioanalyzer (Agilent Technologies, Santa Clara, CA, USA), and then sequenced at the Beijing Genomics Institute (BGI) by Illumina HiSeq 4000 platform (Illumina, San Diego, CA, USA) on a 100‐bp paired‐end run.

### RNA‐seq data analysis

After performing an initial quality assessment, filtered data were first mapped to human reference genome (GRCh37/hg19). Reads were aligned using the following parameters: bwa mem‐T 19. For predicting circRNAs, this study used the ciri software and gencodeV19.annotation.gtf (https://www.gencodegenes.org) as the annotation file. To identify circRNAs in published circRNAs with circBase (which was downloaded from http://www.circbase.org/), we normalized circRNAs as the number of backsplice junction spanning transcripts per million raw reads, and Pearson's correlation analysis was applied to measure the significance of the correlation of expression between each gene pair. Significantly differentially expressed genes were determined by NOISeq and PossionDis, respectively. After filtering out circRNAs with very low counts, circRNAs having probability >0.8 in NOISeq and false discovery rate <0.001 in PossionDis were selected as having significant differential expression.

### Validation of candidate circRNAs

Total RNA and gDNA were subjected to PCR amplification using specific primers annealing at the distal ends of the candidate circRNAs. The amplified PCR products were separated using 2% (w/v) agarose gel and were confirmed by Sanger sequencing. The primer sequences are listed in Table S1. qRT‐PCR was performed using GoTaq® qPCR Master Mix (A6002; Promega, Madison, WI, USA). Melt curve analysis was carried out after the PCR to confirm primer specificity, and the relative level of each circRNA was calculated using the $2^{‐\Delta \Delta C_{t}}$ method.

### Cell line and culture

The SCL‐1 cells (human squamous carcinoma cell line) and TE354.T, HSC‐1 and A2058 cell lines were maintained in our laboratory. The TE353.sk (human normal skin cell line) and A431cell lines were purchased from the Cell Bank of Type Culture Collection of Chinese Academy of Sciences (Shanghai, China). The cell lines were cultured in Dulbecco's modified Eagle's medium (DMEM; Gibco, Grand Island, NY, USA) containing 10% FBS, 100 U·L^−1^ penicillin and 100 µg·mL^−1^ streptomycin in an incubator with 5% CO_2_ at 37 °C.

### Silence and overexpression of candidate circRNAs

The siRNAs targeting candidate circRNAs were synthesized by GenePharma (Shanghai, China). The sequences were listed as follows: si‐hsa_circ_0001360‐1, 5′‐CAGCTACTCAGATCAACCT‐3′ (sense) and 5′‐AGGTTGATCTGAGTAGCTG‐3′ (antisense); si‐hsa_circ_0001360‐2, 5′‐TCAGATCAACCTCTCCACT‐3′ (sense) and 5′‐AGTGGAGAGGTTGATCTGA‐3′ (antisense). The sequence of hsa_circ_0001360 was synthesized and inserted into the EcoRI and BamHI sites in lentiviral vector (pCDH‐CMV‐MCS‐EF1‐Puro) to generate the Lv300‐hsa_circ_0001360 construct, which was confirmed by DNA sequencing (Sangon Biotech, Shanghai, China). The empty lentiviral vector was used as a negative control. The siRNAs and plasmids were transiently transfected into SCL‐1 cells using Lipofectamine 3000 (Invitrogen, Carlsbad, CA, USA) according to the manufacturer's instructions.

### Cell viability and apoptosis assays

One hundred microliters of transfection SCL‐1 cells (4 × 10^4^ cells) was seeded into 96‐well plates and cultured at 37 °C in a 5% CO_2_ incubator for 24, 48 and 72 h. At each time point, 10 µL MTS solution was added to each well, and the plates were incubated for 30 min. Cell viability was measured using spectrophotometer (Hitachi, Tokyo, Japan) at 492‐nm wavelength. For apoptosis assay, the transfected SCL‐1 cells were washed twice with PBS, then were collected and adjusted for a concentration of 10^6^ cells·mL^−1^ by binding buffer. One hundred‐microliter cell suspensions were incubated with 5 µL annexin V/allophycocyanin (APC) and 5 µL 7‐aminoactinomycin D (7‐AAD) at room temperature for 30 min (kept protected from light). Apoptosis was assessed by flow cytometry.

### Colony formation assay

The transfected SCL‐1 cells were collected and adjusted for a concentration of 1 × 10^3^ cells·mL^−1^, and then a total of 50‐, 100‐ and 200‐µL cell suspensions were respectively seeded in six‐well plates with 2 mL medium and cultured at 37 °C for 14 days. Cells were fixed with 4% polyoxymethylene and stained with 200 µL crystal violet; then cells were rinsed by PBS. The cells were photographed and counted under the microscope.

### Cell migration and invasion assay

For cell migration assay, the transfected SCL‐1 cells were suspended with 100 µL serum‐free DMEM (1 × 10^6^ cells·mL^−1^) and seeded into the 8‐μm upper chambers of 24‐well plates. The lower chamber was added with 600 µL DMEM containing 10% FBS. After incubation at 37 °C with 5% CO_2_ for 24 h, cells remaining on the upper side of the filter were wiped off using a cotton‐tipped swab. The cells that had traversed to the bottom surface of the filter were fixed by 4% paraformaldehyde and stained with 1% crystal violet solution. The number of cells was counted based on five fields randomly selected from digital images. To assess invasive ability, transwell chambers were precoated with Matrigel (Corning, New York, NY, USA) according to the manufacturer's protocol. Other procedures were performed as described in the migration assay.

### Prediction of circRNA–miRNA–mRNA interaction network of hsa_circ_0001360

The targeted miRNAs of hsa_circ_0001360 were predicted by TargetScan (http://www.targetscan.org/) and miRanda (www.microrna.org/). The information of miRNA–mRNA regulatory relationships was predicted by the miRTarBase database (http://mirtarbase.mbc.nctu.edu.tw/php/index.php). cytoscape (http://www.cytoscape.org/) was applied to diagram the potential map of the circRNA–miRNA–mRNA interaction network of hsa_circ_0001360. The predicted functions of the hsa_circ_0001360 were annotated using GO (http://geneontology.org/) and KEGG (http://www.genome.jp/kegg/) pathway analysis.

### Statistical analysis

Statistical analyses were performed using spss version 13.0 (SPSS, Chicago, IL, USA). Data are expressed as the mean value ± standard deviation. Difference within two groups was analyzed using Student's *t*‐test or one‐way ANOVA or Pearson chi‐square test. The threshold value used to screen differentially expressed circRNAs and mRNA was a fold change ≥2.0. All experiments were performed in triplicate, and *P* values <0.05 were considered statistically significant.

## Conflict of interest

The authors declare no conflict of interest.

## Author contributions

PC and CL performed the experiments and drafted the manuscript. HH performed the bioinformatics analysis. LL, JZ, QL, and QW collected the clinical data. SZ helped revise the manuscript. JL, KZ and XZ were responsible for study conception and design. All authors read and approved the final manuscript.

## Supporting information


**Table S1.** The sequences of the primers used for PCR, Q‐PCR and Sanger sequencing.

## Data Availability

The RNA‐seq reads from six tissues have been deposited in the NCBI Sequence Read Archive (SRA) with the BioProject number PRJNA701842.
