# Supplementary material for: Circular RNA profiles and the potential involvement of down‐expression of hsa_circ_0001360 in cutaneous squamous cell carcinogenesis
Source: FEBS Open Bio. 2021 Mar 11;11(4):1209–22. doi: 10.1002/2211-5463.13114 (PMC8016141; doi:10.1002/2211-5463.13114)
Supplement: Supplementary file 1 — Table S1. The sequences of the primers used for PCR, Q‐PCR and Sanger sequencing. [file FEB4-11-1209-s001.docx]

Supplementary Table 1 The sequences of the primers used to PCR, Q-PCR and Sanger sequencing.

| Primer name | Sequence (5'-3') | Product size (bp) |
| --- | --- | --- |
| GAPDH-circ-F | TCCTCACAGTTGCCATGTAGACCC | 220 |
| GAPDH-circ-R | TGCGGGCTCAATTTATAGAAACCGGG |  |
| GAPDH-line-F | GAGTCAACGGATTTGGTCGT | 185 |
| GAPDH-line-R | GACAAGCTTCCCGTTCTCAG |  |
| hsa_circ_0018168-circ-F | TCAACGCTAGACCTCAGAGC | 143 |
| hsa_circ_0018168-circ-R | ACCCGTTTTCTCAATCTTGTCA |  |
| hsa_circ_0018168-line-F | AAGCAGGAGCAGGAGAGGAT | 173 |
| hsa_circ_0018168-line-R | GGTCTAGCGTTGAGAGCCAT |  |
| hsa_circ_0000567-circ-F | AACACAGCTCGACAGTACGC | 121 |
| hsa_circ_0000567-circ-R | TCCTTTGGTGACACAGTTGC |  |
| hsa_circ_0000567-line-F | ATGGGCCTCTGAAAATGGGG | 264 |
| hsa_circ_0000567-line-R | CAGAAGGAGTTAGGGCTGGC |  |
| hsa_circ_0000932-circ-F | TTCTTTCTCACTGCCCGC | 103 |
| hsa_circ_0000932-circ-R | GAAGATGATGACCGACCC |  |
| hsa_circ_0000932-line-F | TAGACGAAGAGCGGCGGA | 162 |
| hsa_circ_0000932-line-R | CCTCGTCCAGAATGAAGCCT |  |
| hsa_circ_0001360-circ-F | TTTCACACCCGCTACCACTG | 150 |
| hsa_circ_0001360-circ-R | GAAGCCTGGGAACGGCTTAT |  |
| hsa_circ_0001360-line-F | ATAAGCCGTTCCCAGGCTTC | 96 |
| hsa_circ_0001360-line-R | TTGGCTTGCCGTTAGGGTAG |  |
